# Taxonomic Diversity Not Associated with Gross Karyotype Differentiation: The Case of Bighead Carps, Genus *Hypophthalmichthys* (Teleostei, Cypriniformes, Xenocyprididae)

**DOI:** 10.3390/genes11050479

**Published:** 2020-04-28

**Authors:** Alexandr Sember, Šárka Pelikánová, Marcelo de Bello Cioffi, Vendula Šlechtová, Terumi Hatanaka, Hiep Do Doan, Martin Knytl, Petr Ráb

**Affiliations:** 1 Laboratory of Fish Genetics, Institute of Animal Physiology and Genetics, Czech Academy of Sciences, Rumburská 89, 277-21 Liběchov, Czech Republic; 2 Departamento de Genética e Evolução, Universidade Federal de São Carlos, Rod. Washington Luiz km 235 cep, São Carlos 13565-905, Brazil; 3 Research Institute of Aquaculture No. 1, Dinh Bang, Tu Son, Bac Ninh 16000, Vietnam; 4 Department of Cell Biology, Faculty of Science, Charles University, Viničná 7, 2-128-43 Prague, Czech Republic

**Keywords:** comparative fish cytogenetics, cytotaxonomy, chromosome banding, East Asian cypriniform fishes, FISH, rDNA, snDNA

## Abstract

The bighead carps of the genus *Hypophthalmichthys* (*H. molitrix* and *H. nobilis*) are important aquaculture species. They were subjected to extensive multidisciplinary research, but with cytogenetics confined to conventional protocols only. Here, we employed Giemsa-/C-/CMA_3_- stainings and chromosomal mapping of multigene families and telomeric repeats. Both species shared (i) a diploid chromosome number 2*n* = 48 and the karyotype structure, (ii) low amount of constitutive heterochromatin, (iii) the absence of interstitial telomeric sites (ITSs), (iv) a single pair of 5S rDNA loci adjacent to one major rDNA cluster, and (v) a single pair of co-localized U1/U2 snDNA tandem repeats. Both species, on the other hand, differed in (i) the presence/absence of remarkable interstitial block of constitutive heterochromatin on the largest acrocentric pair 11 and (ii) the number of major (CMA_3_-positive) rDNA sites. Additionally, we applied here, for the first time, the conventional cytogenetics in *H. harmandi*, a species considered extinct in the wild and/or extensively cross-hybridized with *H. molitrix*. Its 2*n* and karyotype description match those found in the previous two species, while silver staining showed differences in distribution of major rDNA. The bighead carps thus represent another case of taxonomic diversity not associated with gross karyotype differentiation, where 2n and karyotype structure cannot help in distinguishing between genomes of closely related species. On the other hand, we demonstrated that two cytogenetic characters (distribution of constitutive heterochromatin and major rDNA) may be useful for diagnosis of pure species. The universality of these markers must be further verified by analyzing other pure populations of bighead carps.

## 1. Introduction

The bighead carps of the genus *Hypophthalmichthys* (Bleeker, 1860) represent a small, well-defined group of morphologically highly distinct and ecologically unique cyprinoid fishes [1] formerly recognized as cyprinid subfamily Hypophthalmichthiynae. Recent formal taxonomy includes this genus into family Xenocyprididae (sensu [2]) and, at the same time, the genus is a member of monophyletic clade harboring several East Asian morphologically distinctly differentiated genera [3]. Collectively, the bighead carps once consisted of monotypic genus *Aristichthys* (Oshima, 1919) with species *Aristichthys nobilis* (Richardson, 1844) (bighead carp) and genus *Hypophthalmichthys* (Bleeker, 1860) with two recognized species: silver carp, *Hypophthalmichthys molitrix* (Valenciennes, in Cuvier & Valenciennes, 1844) and Harmand’s silver carp (or large-scaled silver carp), *Hypophthalmichthys harmandi* (Sauvage, 1884). However, Howes [1] synonymized the genus *Aristichthys* with *Hypophthalmichthys* based on morphological characteristics—a taxonomic action not always accepted [4]. The systematic status of *H. harmandi* is not well understood at present, and while some authors [4] recognized it as a species distinct from *H. molitrix*, others [5] consider it as subspecies of silver carp only; nevertheless, both species differ in a number of morphological, physiological and reproductive characters (for details, see Supplementary File 1: Text S1). 

In their native range (from Amur R. in the north to the Red R. basin in Vietnam and Hainan Island in the south) and elsewhere in temperate regions in Eurasia, they are highly economically important fishes as objects of both lacustrine and riverine fishery and aquaculture [6]. However, bighead carps have been introduced and/or stocked into rivers and lakes outside their native range such as, e.g., in North America (see [7] and references therein), India [8], South Africa [9], and elsewhere in a number of countries [10], where they consequently became invasive aliens which degraded aquatic ecosystems, changing significantly the food webs (see, e.g., in [10,11,12,13,14]). Bighead carps have been and still are objects of intense investigation in various types of studies; for instance, search on 25 April 2020 shows 1155 records on Web of Science and ~19,200 records on Google Scholar when using the term ‘Hypophthalmichthys’. Similarly, the chromosomes of bighead and silver carp have been studied by relatively high number of authors (reviewed in Table 1), although mostly just at the level of conventionally Giemsa-stained chromosomes. 

All those studies identically reported 2*n* = 48 but differed markedly in the karyotype description, evidently due to the low quality of chromosome preparations, except the reports of Liu [26,30] where mitotic chromosomes from the leukocyte cultures were successfully prepared. Only a few of those studies tried to investigate some other chromosomal characteristics using silver staining of nucleolar organizer regions (NORs; Ag-NOR technique) [27], C-banding [27,34], G-banding [35], or BrdU replication banding [33], all with very ambiguous and not reliable results except the one of Almeida-Toledo et al. [27] who evidenced multiple NOR regions on chromosomes of both bighead carp species. However, the chromosomes of *H. harmandi* have not been studied as yet. 

Aiming to more deeply examine the karyotype organization in *H. molitrix* and *H. nobilis*, we combined conventional cytogenetics (Giemsa-, C-, and CMA_3_- stainings) with the chromosomal mapping of 5S and 18S rDNA, U1 and U2 snDNA, and (TTAGGG)*_n_* tandem repeats. In addition, we have undertaken Giemsa karyotyping and Ag-NOR analysis in a third species, *H. harmandi*, which is considered extinct in the wild and/or extensively cross-hybridized with *H. molitrix*. We analyzed individuals of *H. harmandi* from a unique gene pool strain, not hybridized with silver carp.

## 2. Material and Methods

### 2.1. Sampling

We analyzed four juveniles of *H. molitrix* and five juveniles of *H. nobilis* originated from Fishery Farm, Pohořelice, Czech Republic. The geographical origin of the stock of the former is unknown (original brood fishes were imported from Hungary), while the stock of the latter has been derived from imports from U.S.S.R., which have originated in Amur R. Nine juveniles of *H. harmandi* belonged to a pure line maintained at the Research Institute of Aquaculture No. 1, Dinh Bang, Tu Son, Bac Ninh, Vietnam; it originates from Red River in Vietnam and has been derived from the wild population in the late 1950s, i.e., before silver carp introductions from China. These fishes were imported into the Laboratory of Fish Genetics in 1991. Individuals of *H. molitrix* and *H. nobilis* used for the cytogenetic analysis were tested biochemically to confirm the species identity according to the method of Šlechtová et al. [36], who found species-specific alleles in eight allozyme loci. As the analyzed fishes were juveniles, the sex could not be determined. Samples came from the Czech Republic (Petr Ráb) and Vietnam (Hiep Do Doan) in accordance with the national legislation of the countries concerned. To prevent fish suffering, all handling of fish by collaborators followed European standards in agreement with §17 of the Act No. 246/1992 coll. The procedures involving fish were also supervised by the Institutional Animal Care and Use Committee of the Institute of Animal Physiology and Genetics CAS, v.v.i., the supervisor´s permit number CZ 02361 certified and issued by the Ministry of Agriculture of the Czech Republic. All fishes were euthanized using 2-phenoxyethanol (Sigma-Aldrich, St. Louis, MO, USA) before being dissected.

### 2.2. Chromosome Preparation and Conventional Cytogenetics

Chromosome preparations were produced using leukocyte cultures in the case of juveniles of *H. molitrix* and *H. nobilis* [37,38], while those of *H. harmandi* were achieved by a direct preparation from the cephalic kidney [39,40]. The quality of chromosomal spreading was enhanced by a dropping method described by Bertollo et al. [40]. Chromosomes were stained with 5% Giemsa solution (pH 6.8) (Merck, Darmstadt, Germany) for a conventional cytogenetic analysis or kept unstained for other methods. For sequential stainings, selected Giemsa-stained slides were distained in a cold fixation with methanol: acetic acid 3:1 (v/v) before the application of other technique. For FISH, slides were dehydrated in an ethanol series (70, 80, and 96%, 3 min each) and stored at −20 °C. 

Constitutive heterochromatin was visualized by C-banding according to Haaf and Schmid [41]; chromosomes were counterstained with 4′,6-diamidino-2-phenolindole (DAPI) (Sigma-Aldrich). Fluorescence staining was done by GC-specific fluorochrome Chromomycin A_3_ (CMA_3_) and AT-specific fluorochrome DAPI (both Sigma-Aldrich), following Mayr et al. [42] and Sola et al. [43]. The banding protocols were performed either separately or sequentially on the metaphases previously treated by other method(s). In *H. harmandi,* only silver-nitrate impregnation of NORs (i.e, Ag-NOR staining) was performed, according to Howell and Black [44].

### 2.3. DNA Isolation and Preparation of FISH Probes

Total genomic DNA was extracted from fin and blood tissue using the Qiagen DNeasy Blood & Tissue Kit (Qiagen, Hilden, Germany). 5S and 28S rDNA fragments were obtained by polymerase chain reaction (PCR) with primers and thermal profiles described in Sember et al. [45]. Amplification of 18S rDNA and U1 snDNA was done by PCR with the primers 18SF (5′-CCGAGGACCTCACTAAACCA-3′) and 18SR (5′-CCGCTTTGGTGACTCTTGAT-3′) [46]; U1F (5′-GCAGTCGAGATTCCCACATT-3′) and U1R (5′-CTTACCTGGCAGGGGAGATA-3′) [47], using the thermal profiles described in Yano et al. [48] and Silva et al. [47], respectively. The obtained PCR products were purified using NucleoSpin Gel and PCR Clean-up (Macherey-Nagel GmbH, Düren, Germany) according to manufacturer’s instructions. The subsequent procedures involving cloning of the purified products and a plasmid isolation, sequencing (in both strands) of selected positive clones, assembly of chromatograms from obtained sequences and sequence alignment followed essentially the same workflow as described in Sember et al. [49]. Some portion of obtained products was sequenced (in both strands) by Macrogen company (Netherlands). The content of resulting consensus sequences was verified using NCBI BLAST/N analysis [50] and selected clones were used for a FISH probe preparation. For the chromosomal mapping of U2 snDNA, we used the probe obtained previously from a botiid fish *Leptobotia elongata* (for details, see Sember et al. [49]). Furthermore, the FISH results from the mapping of *Hypophthalmichthys*-derived 28S rDNA probe were verified by 28S rDNA probes generated from the nemacheilid loach *Schistura corica* [45] and botiid loach *Botia almorhae* [49]. 

DNA probes were labeled mostly by PCR, either with biotin-16-dUTP or with digoxigenin-11-dUTP (both Roche, Mannheim, Germany). Due to its long size, the 18S rDNA probe was generated in two steps: (i) non-labeling PCR amplification from a verified 18S rDNA clone and (ii) nick translation (2 h) of the amplified 18S rDNA product using Nick Translation Mix (Abbott Molecular, Illinois, USA). A portion of U1 and U2 snDNA probes was also labeled by Nick Translation Mix (Abbott Molecular); the template DNA was in this case the entire plasmid DNA containing U1 or U2 snDNA insert. A dual-color FISH for each slide involved 200 ng of each probe and 25 µg of sonicated salmon sperm DNA (Sigma-Aldrich). The final hybridization mixtures were prepared according to Sember et al. [45]. 

### 2.4. FISH Analysis

Dual-color FISH experiments were conducted essentially according to Sember et al. [45]. Briefly, chromosome preparations were thermally aged (overnight at 37 °C and 1 h at 60 °C), then pre-treated in RNase A (200 µg/mL in 2× SSC, 60–90 min, 37 °C) (Sigma-Aldrich) and pepsin (50 µg/mL in 10 mM HCl, 3 min, 37 °C), and finally denatured in 75% formamide in 2× SSC (pH 7.0) (Sigma-Aldrich) for 3 min at 72 °C. Probes were denatured at 86 °C for 6 min, cooled on ice, and dropped on the chromosome slides. Hybridization took place in a moist chamber at 37 °C overnight. A post-hybridization washing was done under high stringency, i.e., two times in 50% formamide/2× SSC (42 °C, 10 min) and three times in 1× SSC (42 °C, 7 min). Prior to the probe detection, 3% bovine serum albumin (BSA) (Vector Labs, Burlington, Canada) in 0.01% Tween 20/ 4× SSC was applied to the slides to block unspecific binding of antibodies. Hybridization signals were detected by Anti-Digoxigenin-FITC (Roche; dilution 1:10 in 0.5% BSA/PBS) and Streptavidin-Cy3 (Invitrogen Life Technologies, San Diego, CA, USA; dilution 1:100 in 10% NGS (normal goat serum)/PBS). Experiments with altered labeling (e.g., biotin for 18S and digoxigenin for 5S rDNA) were included to verify the observed patterns. All FISH images presented here have a unified system of pseudocolored signals—red for the 18S rDNA and U2 snDNA probes, and green for the 5S rDNA and U1 snDNA probes. Finally, all FISH slides were mounted in antifade containing 1.5 μg/mL DAPI (Cambio, Cambridge, UK).

Telomeric (TTAGGG)*_n_* repeats were detected by FISH using a commercial telomere PNA (peptide nucleic acid) probe directly labeled with Cy3 (DAKO, Glostrup, Denmark) according to the manufacturer’s instructions, with a single modification concerning the prolonged hybridization time (1.5 h).

### 2.5. Microscopic Analyses and Image Processing

Giemsa-stained chromosomes and FISH images were inspected using a Provis AX70 Olympus microscope equipped with a standard fluorescence filter set. FISH images were captured under immersion objective 100× with a black and white CCD camera (DP30W Olympus) for each fluorescent dye separately using DP Manager imaging software (Olympus). The same software was used to superimpose the digital images with the pseudocolors. Karyotypes from Giemsa-stained chromosomes were arranged in Ikaros (Metasystems) software. Final images were optimized and arranged using Adobe Photoshop, version CS6.

At least 15 metaphases per individual and method were analyzed, some of them sequentially. Chromosomes were classified according to Levan et al. [51], but modified as m—metacentric, sm—submetacentric, st—subtelocentric, and a—acrocentric, where st and a chromosomes were scored as uniarmed to calculate NF value (Nombre Fondamental, number of chromosome arms sensu Matthey [52]). Chromosome pairs were arranged according to their size in each chromosome category.

## 3. Results

### 3.1. Karyotypes and Chromosome Banding Characteristics

**Figure 1:**
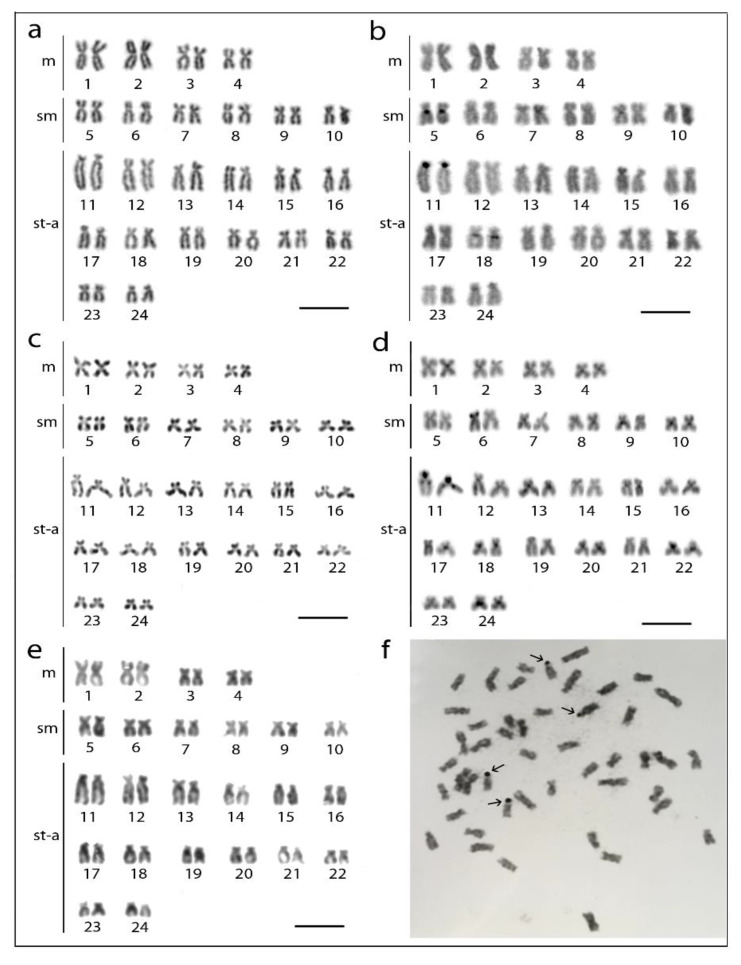
Karyotypes of three *Hypophthalmichthys* species arranged from mitotic metaphases after Giemsa staining, C-banding or Ag-NOR staining. (**a**,**b**) *H. molitrix* (individual HM3), (**c**,**d**) *H. nobilis* (individual HN4), and (**e**) *H. harmandi* (individual HH1). (**a**,**c**,**e**) Giemsa staining; (**b**,**d**) C-banding. Note two distinct blocks of constitutive heterochromatin on pair No. 11 in *H. nobilis* (**d**). (**f**) Ag-NOR staining in *H. harmandi* (individual HH3). The metaphase is incomplete (one chromosome missing; 2*n* = 47), but the most representative one regarding the spreading quality and the signal strength and it is also to higher extent sufficient enough to present required features (i.e., note a lack of Ag-NOR signal on the largest acrocentric chromosome pair No. 11). Scale bar = 10 µm.

**Figure 2:**
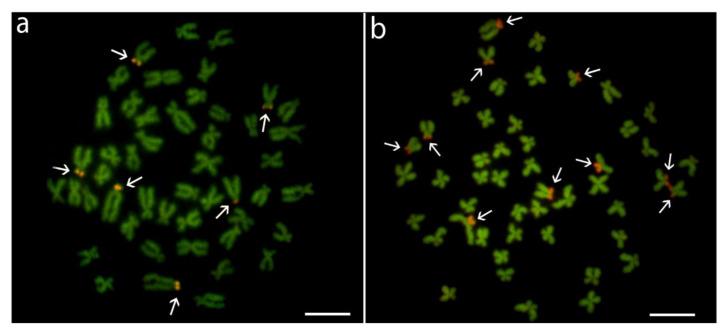
Mitotic metaphases of *Hypophthalmichthys molitrix* and *H. nobilis* after CMA_3_/DAPI staining. (**a**) *H. molitrix*, individual HM3, (**b**) *H. nobilis*, individual HN4. For better contrast, images were pseudocolored in red (for CMA_3_) and green (for DAPI). Arrows indicate CMA_3_-positive sites. Scale bar = 10 µm.

Analyzed fishes of all three species possessed invariably a 2*n* = 48 (Figure 1a,c,e), confirming thus previous reports (Table 1). Besides, they also possessed the same karyotype compositions: four pairs of m, 12 pairs of sm, and eight pairs of st-a chromosomes (Figure 1). Chromosomes of *H. molitrix* and *H. nobilis* displayed a very low content of constitutive heterochromatin concentrated in the pericentromeric chromosome regions, except for significantly heterochromatinized short (*p*) arms of the largest st chromosome pair in *H. molitrix* and additional interstitial block of heterochromatin on this pair in *H. nobilis* only (Figure 1b,d). CMA_3_ fluorescence revealed six positive signals in the karyotype of *H. molitrix* (*p*-arms of the largest and middle-sized st chromosome pairs; Figure 2a), while it displayed altogether 10 signals in *H. nobilis* (all in *p*-arms of st chromosome pairs including the largest st element; Figure 2b). In the karyotype of *H. harmandi*, four Ag-positive signals in the *p*-arms in st chromosome pairs (likely Nos. 17 and 18) were observed (Figure 1f).

### 3.2. Sequence Analysis of Repetitive DNA Fragments

PCR amplification resulted consistently in approximately 150 bp (U1 snDNA), 200 bp (5S rDNA), 300 bp (28S rDNA), and 1800 bp (18S rDNA) long fragments. Searches with the BLAST/N program at NCBI yielded the following results; 18S rDNA (*H. molitrix*)—sequenced 1380 bp long part showed 96–99% identity with 18S rDNA fragments of many fish species; 28S rDNA (both from *H. molitrix* and *H. nobilis*) displayed high similarity results (96–98% identity) with 28S rDNA sequences of many teleosts; 5S rDNA (both from *H. molitrix* and *H. nobilis*): 176–178 nt of our sequenced fragment was subjected to BLAST/N and showed 87–88% identity with sequence of 5S rDNA and non-transcribed spacer of *Megalobrama amblycephala* (Sequence ID: KT824058.1), *Cyprinus carpio* (Sequence ID: LN598602.1) and *Danio rerio* (Sequence ID: AF213516.1), and further 97% identity was shown in 104–114 nt long part of our PCR fragment with the coding region of 5S rDNA of many fishes. Finally, 123 nt of our U1 snDNA fragment showed 97% identity with the predicted U1 snRNA gene region of many fish species. Sequences for 18S rDNA and U1 snDNA (from *H. molitrix*) and for 5S rDNA (from both *H. molitrix* and *H. nobilis*) were deposited in GenBank under the accession numbers MT165584-MT165587. We have not investigated U2 snDNA genes from *Hypophthalmichthys* as the U2 snDNA probe from *Leptobotia elongata* has proven to be fully sufficient for FISH.

### 3.3. Hybridization Patterns of Repetitive DNA Probes

**Figure 3:**
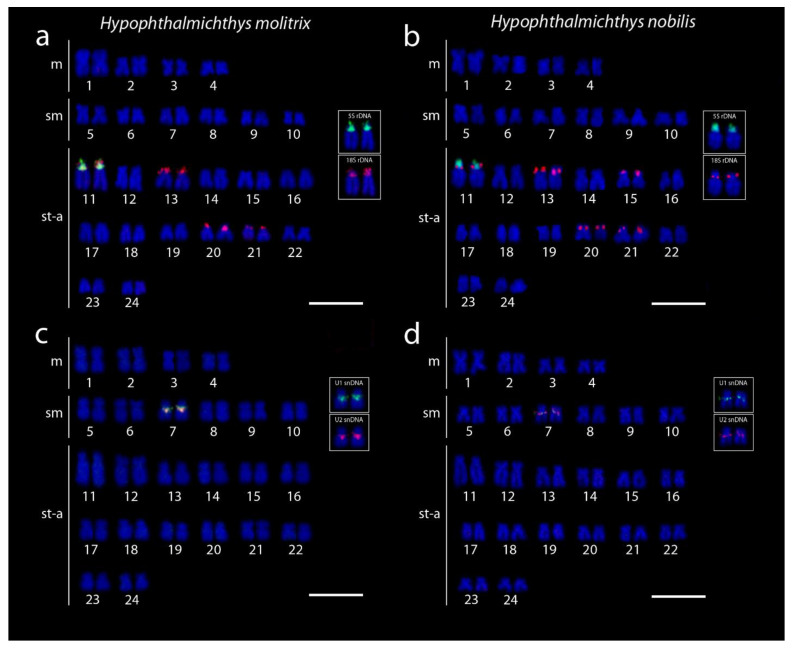
Karyotypes of *Hypophthalmichthys molitrix* and *H. nobilis* arranged after 5S/18S rDNA and U1/U2 snDNA FISH. (**a**,**b**) 18S rDNA (red) and 5S rDNA (green) probes. Insets show separately 5S and 18S rDNA signals on the largest acrocentric pair. Note the adjacent position of 5S and 18S rDNA signals on chromosome pair No. 11 in both species. (**c**,**d**) U1 (green) and U2 (red) snDNA probes mapped on mitotic chromosomes of (**c**) *H. molitrix* and (**d**) *H. nobilis*. Note the co-localization of a single pair of U1 and U2 snDNA signals in small sm chromosome pair No. 7. Insets show separate hybridization signals for each individual probe. Chromosomes were counterstained with DAPI (blue). Identification codes of individuals: (**a**) *H, molitrix* HM2, (**b**) *H. nobilis* HN1, (**c**) *H. molitrix* HM4, and (**d**) *H. nobilis* HN3. Scale bar = 10 µm.

5S rDNA probe mapped consistently to the proximal region of the largest acrocentric pair No. 11 in both species (Figure 3a,b). On the same chromosome pair, adjacent to 5S rDNA cluster, tandem arrays of 18S rDNA were found to cover the entire *p*-arms (Figure 3a,b). Additional 18S rDNA loci resided in the terminal part of *p*-arms or encompassed entire *p*-arms of several chromosomes. The complete number of 18S rDNA signals was eight in *H. molitrix* (chromosome pairs 11, 14, 20, and 21) and ten in *H. nobilis* (chromosome pairs 11, 14, 15, 20, and 21) (Figure 3a,b). On the other hand, 28S rDNA probes (generated from herein studied species or utilized from other cypriniforms formerly analyzed by us [45,49]) did not generate any hybridization signals, suggesting that a 300 bp long probe is too short to visualize small rDNA clusters present in *Hypophthalmichthys*, while 1800 bp of 18S rDNA can produce signals of sufficient intensity. Although all the 18S rDNA sites corresponded with CMA_3_-positive signals, some 18S rDNA clusters in *H. molitrix* were not revealed by this GC-specific fluorochrome (compare Figure 2a and Figure 3a), again probably reflecting small size (i.e., relatively low copy number of tandem arrays) of major rDNA cistrons.

**Figure 4:**
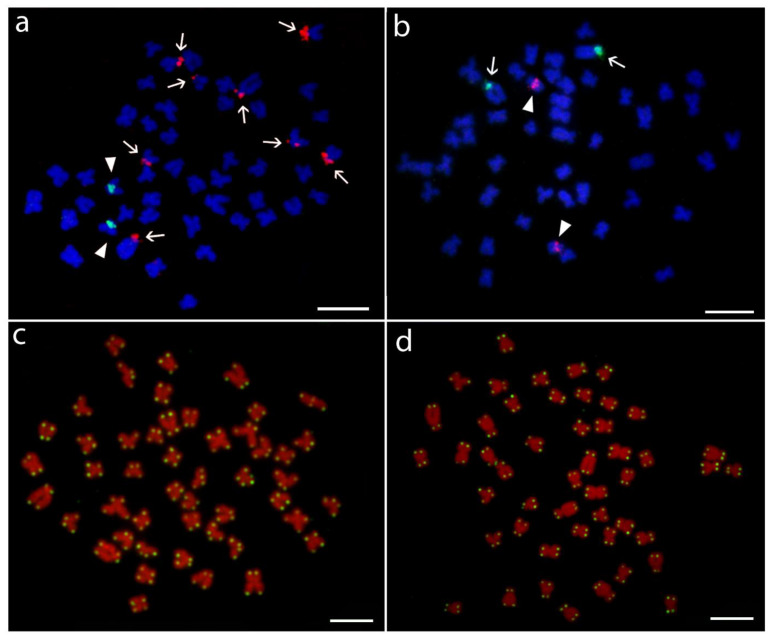
Mitotic metaphases of *Hypophthalmichthys molitrix* and *H. nobilis* after different cytogenetic treatments. (**a**,**c**) *H. molitrix* and individual HM4 in both methods; (**b**,**d**) *H. nobilis*, individuals HN4, and HN3, respectively. Images (**a**,**b**) clarify an independent location of distinct cytogenetic markers. (**a**) FISH with U1 snDNA (green, arrowheads) and 18S rDNA (red, arrows) probes. (**b**) FISH with U2 snDNA (red, arrowheads) and 5S rDNA (green, arows) probes. Chromosomes were counterstained with DAPI (blue). (**c**,**d**) PNA FISH with telomeric probe; for better contrast, pictures were pseudocolored in green (telomeric repeat probe) and red (DAPI). Scale bar = 10 µm.

**Figure 5:**
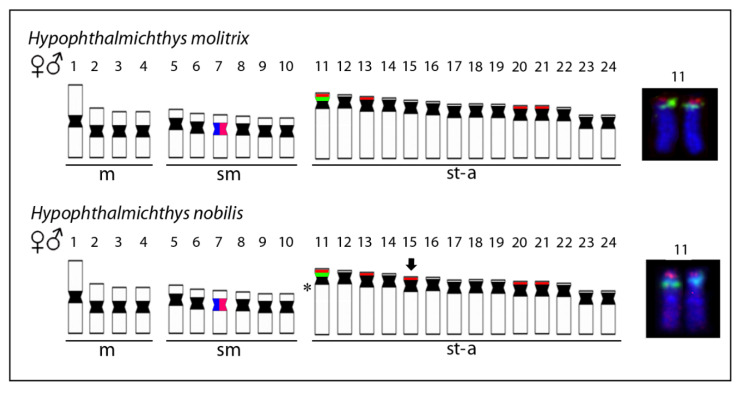
Representative idiograms of two *Hypophthalmichthys* species highlighting the distribution of analyzed multigene families. 18S (red) and 5S (green) rDNA sites and U1 (blue) and U2 (pink) snDNA sites on the chromosomes of *H. molitrix* and *H. nobilis*. Note the co-localization of snDNA sites on the chromosome pair 7 and the adjacent arrangement of 5S and 18S rDNA sites on the chromosome pair 11. Moreover, notice the additional 18S rDNA site on chromosome pair 15 in *H. nobilis* (marked by arrow) in comparison to the karyotype of *H. molitrix*. Finally, an asterisk denotes the location of the differential interstitial C-band, which is present in *H. nobilis* but absent in *H. molitrix*. Insets with the chromosome pair 11 (right) display the chromosomes dissected from prometaphase plates after rDNA FISH, where the adjacent arrangement of both rDNA classes is clearly visible.

U1 and U2 snDNA probes co-localized in both species in a pericentromeric region of small st chromosome pair (No. 7) (Figure 3c,d). Neither the co-localization between snDNA and rDNA (Figure 4a,b and Figure 5), nor intraspecific variability in the number of hybridization signals of any multigene family were observed among analyzed individuals of both species. Telomere FISH marked only ends of all chromosomes, with no additional interstitial sites (Figure 4c,d). 

As we analyzed not sexed juvenile individuals, we could not directly assess possible sex-related differences in the karyotypes and in patterns of analyzed cytogenetic markers. Nonetheless, we did not observe any type of within-species polymorphism in our sampling, and it has been formerly shown that both *Hypophthalmichthys* species display a sex ratio around 1:1 due to genetic sex determination governed most likely by a homomorphic (i.e., cytologically indistinguishable) XX/XY sex chromosome system [53].

## 4. Discussion

The chromosomes of the two species of bighead carps, *H. molitrix* and *H. nobilis*, were extensively studied (Table 1), evidently due to their high aquacultural value. On the other hand, 2*n* and karyotype of the third species of the genus, *H. harmandi*, is reported in our study for the first time. Our current assessment of the karyotype structure and the hybridization patterns of selected multigene families in *H. molitrix* and *H. nobilis* is summarized in Figure 5. Our study confirmed 2*n* = 48 for these two species and revealed the same chromosome count for *H. harmandi*. The karyotype structures in *H. molitrix* and *H. nobilis*, however, differed markedly among various studies. The reason for these discrepancies might be linked with the following facts; (i) chromosomes of cypriniform fishes generally exhibit very small size when compared to other teleosts (see, e.g., in [45,54,55,56]); (ii) furthermore, cyprinoid chromosomes also exhibit a gradual decrease in size, with the centromere positions ranging stepwise from median to nearly terminal, making it difficult to assess the chromosomal categories with accuracy; and (iii) inspection of published chromosome pictures showed that previous reports were based on highly condensed chromosomes which also made it impossible to describe the karyotype accurately. However, careful analysis of a number of metaphase cells with less condensed chromosomes demonstrated that karyotypes of all three species of bighead carps at the level of conventionally Giemsa-stained chromosomes are in fact identical.

We thus show that potential interspecific hybrids between *H. harmandi* and *H. molitrix* cannot be revealed after basic karyotype analysis alone. Nonetheless, we observed that karyotypes of *H. molitrix* and *H. nobilis* differ in two other cytogenetic characters; one of them displays distinctive pattern also in *H. harmandi*. First, the presence of an additional interstitial C-band on the largest acrocentric pair in all individuals of *H. nobilis* clearly distinguishes this species from *H. molitrix,* at least in our sampled populations. This additional location of constitutive heterochromatin in *H. nobilis* might potentially emerge after a pericentric inversion which did not affect the general morphology of the chromosome but relocated part of the heterochromatic block from the *p*-arm to the proximal region of the long (*q*) arm. Our data, however, cannot rule out the involvement of other mechanisms such as centromere repositioning [57,58]. For the third species, *H. harmandi*, data from C-banding are not available; therefore, we cannot confirm if this method alone may provide enough information to discriminate the karyotypes of all three species. Nonetheless, even if we could do that, we would have to take into account that constitutive heterochromatin might display a polymorphic distribution among populations of diverse taxa (including teleosts; exemplified in [59,60,61,62]) and thus this feature might limit the resolution power of C-banding for interspecific diagnosis. Second, we found a difference in the number and position of major rDNA sites—four loci in *H. harmandi*, eight in *H. molitrix* and ten in *H. nobilis*. Our results are partially not consistent with those of Almeida-Toledo et al. [27] who also reported four pairs bearing Ag-NORs in *H. molitrix*, but only three pairs in *H. nobilis* (in contrast to five pairs revealed by us via FISH). Our view on this discrepancy is that either (i) the Ag-NOR method detected only clusters active in preceding interphase, while our FISH analysis showed all major rDNA sites irrespective of their transcriptional activity, or (ii) Almeida-Toledo et al. [27] examined the hybridized individuals which remained undetected due to lack of testing for genome admixtures, i.e., the step that we included in our present study. In either case, both studies collectively suggest that the patterns of major rDNA distribution might be stable at least in *H. molitrix* and that it differs from the one found in *H. nobilis*, strengthening the possibility that this marker may be useful also in species diagnosis in other *Hypophthalmichthys* populations.

Major (45S; NOR-forming) and minor (5S; located outside NOR) rDNA clusters are by far the most utilized cytotaxonomic markers in fishes [63,64,65]. Major rDNA is usually visualized by 18S or 28S rDNA probes. Despite the ever-growing number of studies showing lability of their site number and patterns of distribution in fish genomes (with many cases documenting intra- and inter-populational variability) (see, e.g., in [66,67,68]) and even their vulnerability to change rapidly under different environmental conditions [69] or hybridization [70], certain arrangements of rDNA classes can help to clarify a presence of species complexes or cryptic species (see, e.g., in [71,72,73]), to uncover the genome composition in hybrid specimens [74,75], to confirm the ploidy level, and to deduce the mechanism of polyploidy [76,77,78,79]. It has been repeatedly documented that even closely related species may possess dramatically different number of rDNA loci [45,71,80]. A difference in number of 5S rDNA clusters between emerald and darter goby (two vs. 42) [81] may serve as an illustrative example. Besides the difference in number and position of positive signals, also the linkage between 45S and 5S or them with other multigene families may represent a valuable cytotaxonomic determiner (see, e.g., in [82,83,84,85]). 

Among Cypriniformes, many studies have been conducted on polyploid species and especially on those of high aquacultural importance, such as genera *Cyprinus* and *Carassius* (see, e.g., in [55,74,86,87]) or on unisexually reproducing taxa such as *Squalius*, *Cobitis*, and *Misgurnus* and on species closely related to them [45,77,79,88,89,90,91,92]. Some reports revealed amplified number of either 5S or 18S rDNA signals [45,55], different types of inter-individual/inter-populational polymorphisms in number and location of rDNAs [70,88,89,90,91,93] or high interspecific variability in this character [91,92,94], while still other studies found rather standard patterns, with just one locus of one or both rDNA classes per haploid genome [45,77,95] or only a slight elevation in number of sites [56,95,96]. Among two *Hypophthalmichthys* species analyzed herein, a single pair of 5S rDNA loci occupied apparently homeologous chromosomes and were found adjacent to one of the multiple 18S rDNA clusters. Similar links between 5S and 18S rDNA sites provided valuable cytotaxonomic markers in some cyprinids (see, e.g., in [86,92,96]). In our study, however, as this arrangement is shared by both species, it cannot be considered as useful cytotaxonomic determiner. Nonetheless, Ag-NOR analysis in *H. harmandi* clearly showed that NORs are not present on this largest acrocentric pair, hence potential hybrids containing the *H. harmandi* genome could be identified this way. What is further evident is the interspecific difference in the number of 18S rDNA sites, which could be helpful as a cytotaxonomic marker, but its intraspecific stability must be further verified in other pure populations of both species. In this sense, it may be difficult to discriminate all 18S rDNA loci due to their tiny size, therefore the analysis should be treated with caution.

Genes for small nuclear RNA (snRNA) are yet readily used for chromosome mapping in fishes, though studies employing U2 snDNA as a cytogenetic marker are steadily growing in the last years ([84,97,98,99,100,101], to name a few). On the other hand, U1 snDNA has been so far chromosomally mapped only in a cichlid *Oreochromis niloticus* [102], several South American characiforms of the genera *Astyanax* [47] and *Triportheus* [85], and further in African characiform representative *Hepsetus odoe* [103], one species from Gadiformes [104] and one taxon (suspected species complex) belonging to Mugiliformes [73]. Among cypriniforms, only a single recent work mapped U2 snDNA sites, namely in diploid and tetraploid loaches of the family Botiidae [49], therefore our present study is the first one showing the position of U1 snDNA on cypriniform chromosomes. In botiids, perhaps surprisingly, the mapping of U2 snDNA showed mostly a conserved single pair of U2 snDNA signals irrespective of the ploidy level. What is more, the location of U2 snRNA arrays in the pericentromeric/interstitial region as revealed in botiids was also found herein in both *Hypophthalmichthys* species and, interestingly, the same or similar pattern has been encountered in approximately half of fish species inspected for U2 snDNA distribution to date (see [84,101] and examples listed in Yano et al. [99]). It seems that a strong selective pressure operates to maintain such a location for this gene. Moreover, in botiids [49] as well as in two herein studied *Hypophthalmichthys* species and in some other fish species [47,101,105,106] snDNA clusters are located on rather small-sized chromosomes. It is tempting to hypothesize that this location may facilitate more efficient expression as small chromosomes tend to occupy rather interior, transcriptionally active part of the interphase nucleus (see, e.g., in [107]). What is less conserved, is the so far known association of U2 snDNA with other multigene families. Several combinations of syntenic/adjacent or intermingled arrangements can be found among fishes such as between 5S rDNA and U1 snDNA [47,85], 5S rDNA and U2 snDNA [84,98,100,105], 18S rDNA with U2 snDNA [99,106,108], 5S and 18S rDNA together with U2 snDNA [99] and even with several histone genes [109]; further U1 and U2 snDNA [97,104] or U1 and U2 snDNA together with 5S rDNA [110]. Therefore, these arrangements may potentially serve as useful cytotaxonomic markers. In our study, both investigated *Hypophthalmichthys* species shared the co-localization of U1 and U2 snDNA cistrons along with an independent location of these sites with respect to rDNA classes.

FISH aimed to map the vertebrate telomeric (TTAGGG)*_n_* repeat motif showed signals only in their usual location at termini of all chromosomes. No interstitial telomeric sequences (ITSs), which might point to previous structural chromosomal rearrangements (see, e.g., in [111]), were detected, neither in *H. molitrix* nor in *H. nobilis*. More importantly, this type of analysis did not reveal any differences between analyzed species that would be helpful in their discrimination.

Recently, all three herein studied species are included in the genus *Hypophthalmichthys* (Bleeker, 1860) [1] but Kottelat [4] noted that not all authors agree with synonymization of the genus *Aristichthys* (Oshima, 1919). From the cytotaxonomic view, it is not possible to contribute to this problem due to lack of significant karyotype differences. Table 2 further summarizes all available data for members of the monophyletic East Asian clade of the family Xenocyprididae (sensu Tan and Ambruster [2]). Though the quality of such data was affected by the facts discussed above (i.e., the characteristics of cyprinoid chromosomes), their critical assessment demonstrates that these species possess (i) the same 2*n* = 48; (ii) very similar karyotype structures; and, where studied [112,113], also (iii) multiple NOR sites, supporting thus molecular phylogeny of the clade [3]. 

The stability of 2n (with either 48 or 50 chromosomes) is widely documented for majority of non-polyploid cyprinoids [91,92,95,120] as well as in other related cypriniforms (see, e.g., in [45,89]), indicating its high conservatism. These signs of the so-called karyotype stasis, in which identical or almost identical karyotypes are maintained within a certain taxonomic group even over considerable long evolutionary time, are observable also in other teleost lineages such as in the pikes of the genus *Esox* [121,122], several lineages of salmonid fishes with A-type karyotype [123,124], and further especially in knifefishes of the family Notopteridae (see [125] and references therein) and many percomorph groups [126,127,128,129,130]. Karyotype stasis has been also documented in diverse clades across the tree of life (e.g., typically in birds [131] and in feline lineages [132]). The underlying evolutionary mechanisms for this mode of karyotype evolution have not been identified so far but they may be at least partially linked with the functional arrangement of chromatin within the interphase nucleus and the degree of tolerance to its change [133,134]. Nonetheless, it is highly probable that such a high degree of karyotype similarity may significantly contribute to the rate of interspecific hybridization [135], which has been repeatedly documented among many cyprinids [19,55,70,75] as well as between the *Hypophthalmichthys* species [18,19,20,21,36].

## 5. Conclusions

Our cytogenetic study of all three species of the genus *Hypophthalmichthys* documented that their karyotype macrostructure, i.e., the number of chromosomes in respective morphological categories, is identical, therefore these characteristics alone may not help in the identification of pure species and interspecific hybridizations. A brief overview of available cytogenetic data of other members of the monophyletic clade of East Asian fishes, to which *Hypophthalmichthys* belongs, shows identical 2*n* = 48, very similar karyotypes and, in a subset of analyzed species, also multiple NOR sites, supporting thus the molecular phylogeny of the clade. The bighead carps thus belong to the teleost lineages where the taxonomic diversity is not associated with extensive karyotype repatterning. However, an important difference has been unraveled in the present study between *H. molitrix* and *H. nobilis* as the latter species exhibits additional interstitial band of constitutive heterochromatin on the largest acrocentric pair 11. Lack of data for *H. harmandi* did not allow us to assess the usefulness of this marker in this practically extinct species. On the other hand, a combined set of FISH and Ag-NOR results showed that the karyotypes of all three species differ among each other in the number and position of major rDNA sites—four in *H. harmandi*, eight in *H. molitrix*, and ten in *H. nobilis*. Particularly important is the absence of major rDNA on the largest pair 11 in the karyotype of *H. harmandi*, which may distinguish this species from the other two. Therefore, the combination of both cytogenetic methods may be useful for the species diagnosis inside *Hypophthalmichthys*. Testing of their universality across different pure *Hypophthalmichthys* populations together with concomitant generation of another cytogenetic markers (such as, e.g., species-specific satellite DNA classes) is an inevitable further research step.

## Figures and Tables

**Table 1:** Summary of reported data on diploid chromosome number (2*n*), numbers of chromosomes in particular morphological categories (m—metacentric, sm—submetacentric, st—subtelocentric, a—acrocentric) and number of chromosome arms (NF value).

Species	2*n*	Karyotype Composition				NF	References
		m	sm	st	a		
*H. nobilis*	48	20	16		12	84	[15,16,17,18]
	48	- 26 -		20	2	74	[19]
	48	18	30			96	[20,21]
	48	14	24		10	86	[22,23]
	48	6	36	6		96	[24,25]
	48	26	20	2		96	[26]
	48	- 24 -		24		72	[27]
*H. molitrix*	48	10	- 26 -		12	84	[28]
	48	20	12	6	10	82	[29]
	48	- 20 -		- 28 -		68	[19]
	48	22	14		12	84	[15,16]
	48	14	24		10	86	[22,23]
	48	24	16	8		96	[30]
	48	20	24		4	96	[31]
	48	18	22	8		88	[32]
	48	- 24 -		24		72	[27]
	48	18	22	8		92	[33]

Note: During the search for data on cytogenetics of bighead carps, we found also eight other studies (published between years 1976–1985) but we did not include them in this summary because they provided 2*n* only and/or were found methodically very problematic. Their list is available upon request from the corresponding author.

**Table 2:** Review of reported cytogenetic data for members of the monophyletic clade of several East Asian morphologically distinct genera.

Species	2*n*	Karyotype Composition				NF	References
		m	sm	st	a		
*Elopichthys bambusa*	48	10	24	12	2	82	[112,114]
*Luciobrama microcephalus*	48	12	22	12	2	82	[114]
*Ochetobius elongates*	48	10	16	22		74	[114]
*Squaliobarbus curriculus*	48	14	30		4	92	[114]
*Culter oxycephaloides*	48	20	24		4	92	[115]
*Xenocypris macrolepis*	48	20	26	2		94	[112,115]
*Xenocypris davidi*	48	18	26	4		92	[112,115]
*Xenocypris fangi*	48	16	28	4		92	[112]
*Xenocypris sechuanensis*	48	18	26	4		92	[112]
*Megalobrama amblycephala*	48	18	26	4		92	[115,116,117]
*Megalobrama terminalis*	48	18	22	8		88	[115]
*Ctenopharyngodon idella*	48	18	24	6		90	[115]
	48	18	30			96	[118]
	48	18	22	8		88	[116]
*Mylopharyngodon piceus*	48	14	- 34 -			96	[25]
	48	16	28	4		92	[119]

## Supplementary Materials

The following are available online at https://www.mdpi.com/2073-4425/11/5/479/s1, Supplementary File 1: Text S1. Morphological differences between *Hypophthalmichthys molitrix* and *H. harmandi*, supplemented with a photographical documentation.
