# Supplementary material for: Taxonomic Diversity Not Associated with Gross Karyotype Differentiation: The Case of Bighead Carps, Genus Hypophthalmichthys (Teleostei, Cypriniformes, Xenocyprididae)
Source: Genes (Basel). 2020 Apr 28;11(5):479. doi: 10.3390/genes11050479 (PMC7291238; doi:10.3390/genes11050479)

Supplementary File 1: Text S1. Morphological differences between *Hypophthalmichthys molitrix* and *H. harmandi*

H. molitrix and *H. harmandi* differ in several morphological characters: number of scales in *linia lateralis* (103 – 112 in *H. molitrix* and 84 – 93 in *H. harmandi*), shape of the abdominal keel (one-arched and two-arched, respectively), length of pectoral fins (longer, reaching beyond base of pelvic fins and shorter, not or maximally reaching the base of pelvic fins, respectively), live body color (lighter and finely spotted and darker back never spotted, respectively). Moreover, both species differ in several other characters such as number of eggs (always higher in silver carp), interval to reach sexual maturity (one year in silver carp but always two years in Harmand's silver carp) and different growth rates in the first two years (always faster in silver carp in the first year but always slower in the second). An earlier study documents that both species may differ also biochemically, in allozyme phenotypes [1].

Supplementary Reference

1. Payusova, A.N.; Tselikova, T.N. Genetic structure of the silver carp *Hypophthalmichthys harmandi* (Sauvage) from Vietnam. *Genetika* **1993**, *29*, 1685 – 1696. (in Russian).

Next page: photographs of *H. molitrix* (upper) and *H. harmandi* (lower).

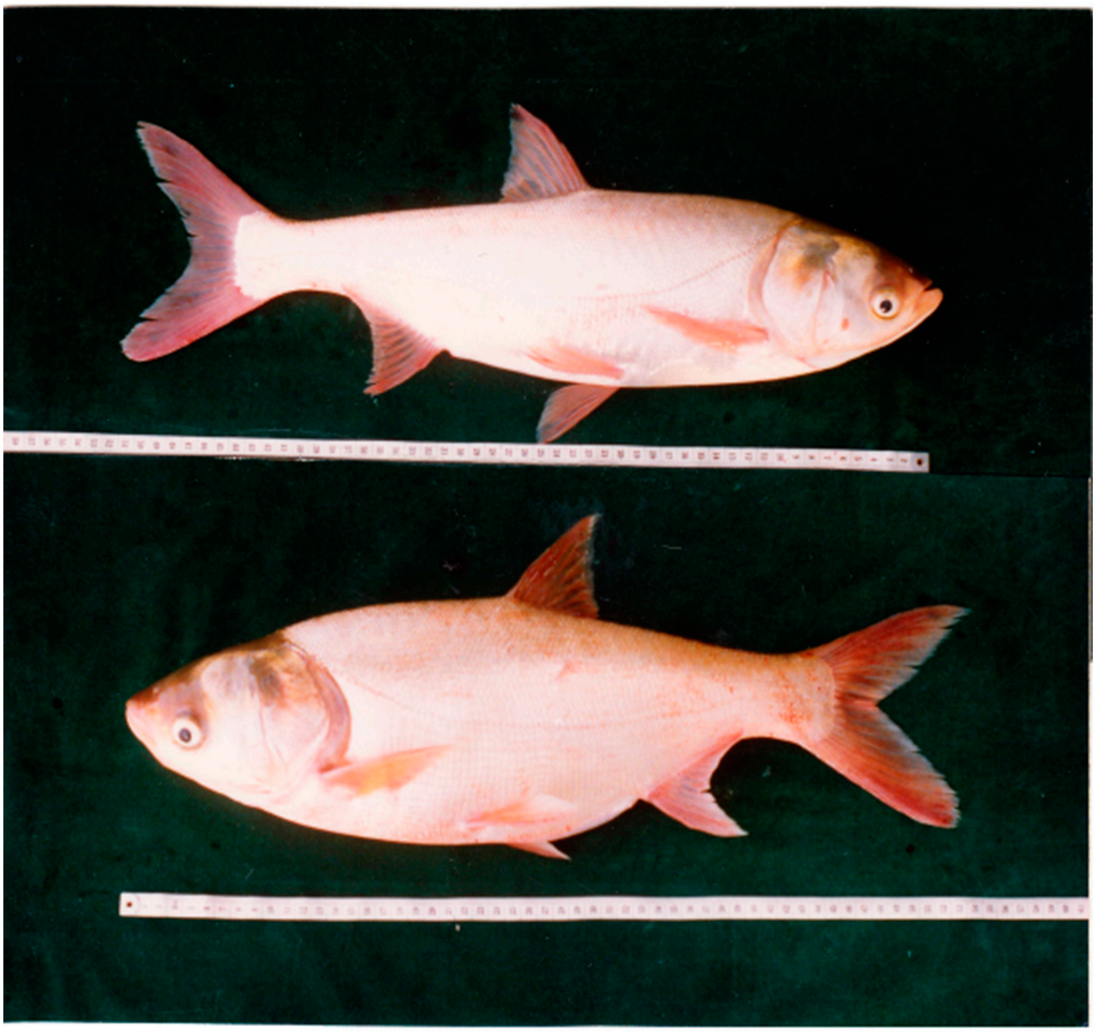

Supplement: Supplementary file 1 [file genes-11-00479-s001.pdf]
